# Estimating carrying capacity and stocking rates of rangelands in Harshin District, Eastern Somali Region, Ethiopia

**DOI:** 10.1002/ece3.5786

**Published:** 2019-11-04

**Authors:** Derege Tsegaye Meshesha, Muhyadin Moahmmed, Dahir Yosuf

**Affiliations:** ^1^ Institute of Pastoral and Agro‐Pastoral Development Studies (IPADS) Jijiga University Jijiga Ethiopia; ^2^ Geospatial Data and Technology Center College of Agriculture and Environmental Science Bahir dar University Bahir dar Ethiopia

**Keywords:** carrying capacity, forage biomass, grazing land, stocking rates, tropical livestock unit

## Abstract

We conducted a quantitative assessment of forage biomass in Harshin district to determine its annual productive potential, carrying capacity, and stocking rates. The dominant Land Use and Land Cover include woodland (35.5%), shrubs (28.3%), grassland (10.6%), and bare land (25.5%). The region has browse‐rich shrubland that is edible to dromedary and goats, as well as massive grassland plains for sheep and cattle. The interannual rainfall variation is 16.5% which implies that the rangeland is a subsistence equilibrium system. The range of forage production is between 105 and 2,310 kg/ha, whereas the average productivity of the district is 742.6 kg/ha. The result indicates that the average carrying capacity of the district is 0.3 TLU ha^−1^ year^−1^ (4.9 ha TLU^−1^ year^−1^) while the existing stocking rate is 5.4 TLU ha^−1^ year^−1^ (0.18 ha TLU^−1^ year^−1^). This implies that the grazing intensity in the district is much higher than its carrying capacity (recommended rate), which has seen overstocking or grazing pressure excesses of 5.1 TLU/ha (7.2 cattle/ha). Thus, it clearly signals the risk of overgrazing in the district. If this trend continues, the grazing will not be sustainable and there will be shortage of forage as well as expansion of land degradation (due to overgrazing) in the near future.

## Graphical abstract

(a) Carrying capacity and stocking rate of Harshin. (b) Measuring forage biomass of Harshin.

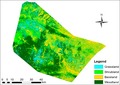

## 1 INTRODUCTION

Livestock rearing is one form of agricultural production which includes dromedaries, cattle, sheep, goats, equines (horses), pigs, and poultry (Jahnke, 1982). The grassland and browse in the pastoral areas of Africa are characterized by low levels of productivity and high variability in yields, both within and across years (De Leeuw & Tothill, 1990; Maass, Musale, Chiuri, Gassner, & Peters, 2012). Evidence from arid environments (e.g., Mongolia, Syria, and Western Australia) with high rainfall coefficients of variability (CV) suggests that these systems are well described by the nonequilibrium model (Engler, Abson, Feller, Hanspach, & Wehrden, 2018; Ward, Ngairorue, Kathena, Samuels, & Ofran, 1998). However, Vetter (2005) reported that most African rangelands have interannual rainfall variability of less than 33%; hence, the management generally followed the principle of equilibrium systems and the assumption that these systems are overstocked and degraded.

Carrying capacity (CC) has widely been used as a tool in rangeland management (Cheng et al., 2017; Tewari & Arya, 2004; Walker, 1995). De Leeuw and Tothill (1990) defined livestock CC as the maximum number of animals (usually expressed as a standardized livestock unit) that an area of land can support on a sustainable basis. It depends upon certain factors such as rainfall, vegetation accessibility and distribution, seasonality, range improvement, and grazing management (Abbas, Saleem, Sharif, & Mirza, 2012; Cheng et al., 2017). Because of so many variables, there is no simple way to quantitatively determine carrying capacity. Yet, it may vary from year to year in the same area due to fluctuating forage production (Christian, 1998).

Carrying capacity has not a fixed value, hence, it is necessary to estimate CC for a given grazing site because it widely varies from place to place (Mulindwa, Galukande, Wurzinger, Okeyo, & Sölkner, 2009). It is important to assess and monitor the condition and CC of a given grazing site in order to understand whether the current management practices are appropriate or require interventions for sustainability (Abbas et al., 2012; De Leeuw & Nyambaka, 1988).

The Ethiopian Somali region, which is located in the Eastern part of Ethiopia, is a typical arid and semiarid area, whose characteristics are similar to many other areas in the Horn of Africa. In the region, the dominant livelihood is pastoralism. It is an extremely fragile environment in which the frequent threats of droughts (e.g., 2015 and 2016) affect not only the survival of livestock but also the human population. However, unlike the highland part of Ethiopia, where rain‐fed agriculture is dominant livelihood, relatively less emphasis is given to this part of the country in research and other developmental activities. One of the main reasons for this is lack of infrastructure and facilities (e.g., roads and accommodations) and poor security conditions due to political instability across the border with mainland Somalia. This discourages researchers and development workers from visiting the area.

**Figure 1:**
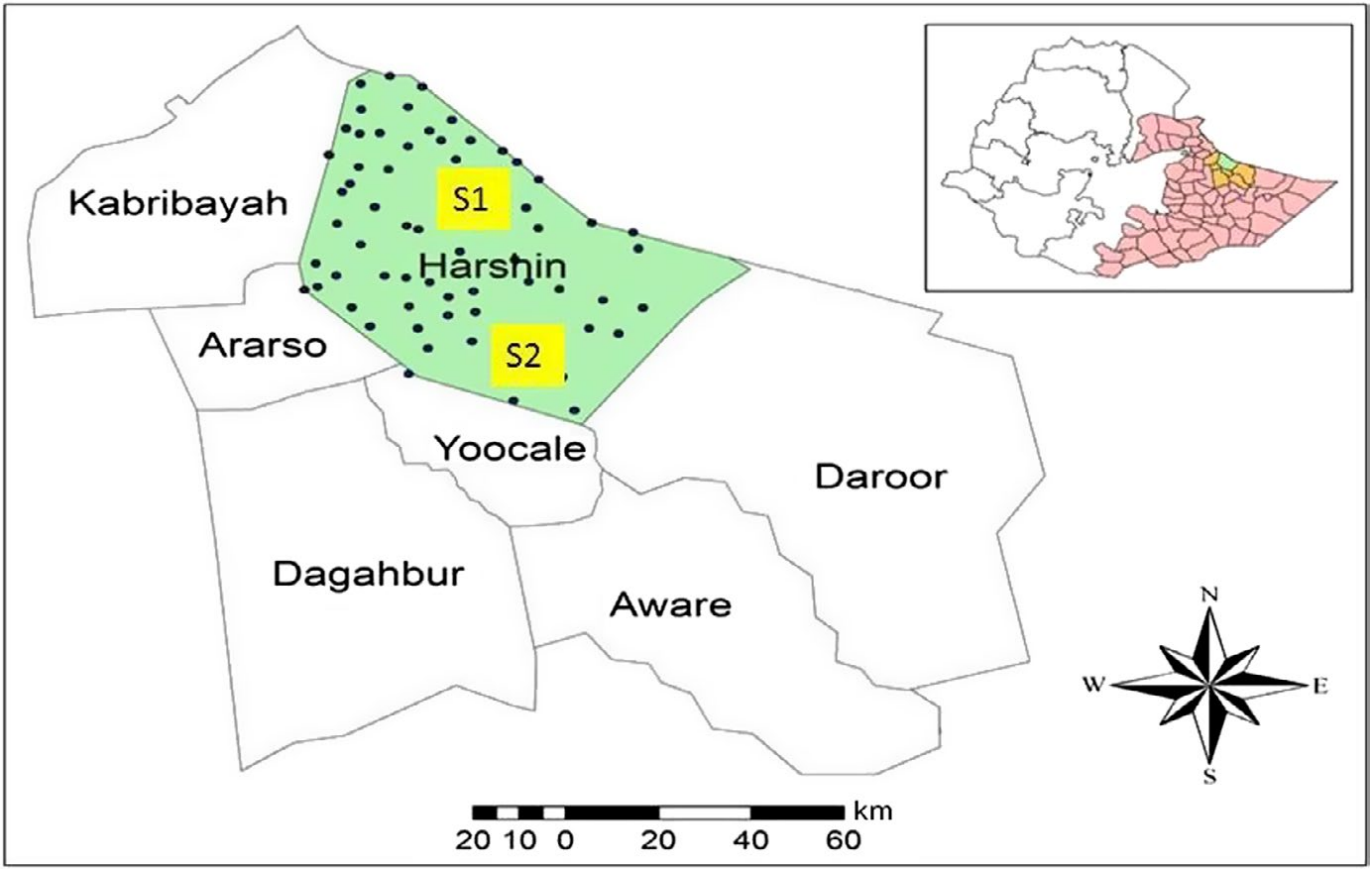
Location map of the study area: Harshin and its neighboring districts in Faafen zone, Eastern Somali region, Ethiopia (S1 and S2 indicates the sites where biomass measurement was conducted)

**Figure 2:**
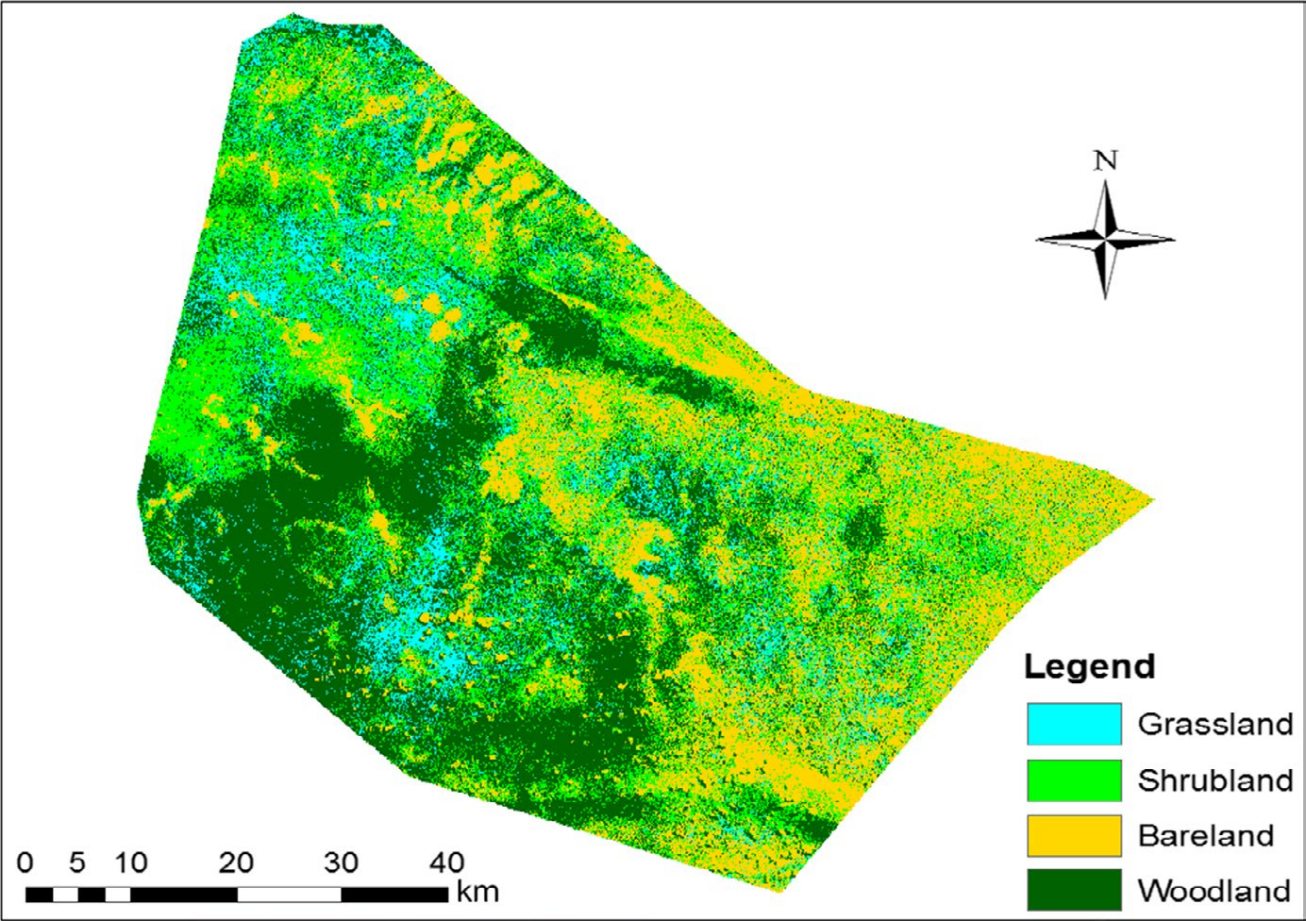
Wider practice of area closure as can be seen from Sentinel image (June, 2018)

Information is very scarce with regard to rangelands condition and their CC in the Somali region, especially in Harshin district (Figure 1). This area has long been under the traditional rotational grazing system but is currently shifting to private area enclosure. This district is an important site in Somali region, which has relatively larger rangeland (dominated by grass and shrubs; Figure 2). However, the current annual production from rangelands is not exactly known so it is difficult to determine stocking rate and CC. On the other hand, determining the actual stocking rate and CC is very essential to recognize the status of rangeland and the balance between animal number and grassland resources (forage performance).

Hence, it is imperative to assess the rangeland condition, stocking rate, and livestock carrying capacity in the area to generate new information about the status of livestock population and vegetation that is necessary for grazing plans. Based on this premise, the research team of the Institute of Pastoralist and Agro‐Pastoralists Development Studies (IPADS) has focused on the understanding of dominant livestock types, existing stocking rates and their spatial variation, potential and actual carrying capacities of Harshin district, Somali region, Ethiopia. Therefore, the main objectives of this study are to (a) characterize and quantify livestock types, (b) estimate the spatial coverage of rangelands, and (c) determine the current biomass production, stocking rates, and carrying capacity of rangelands in Harshin district.

## 2 MATERIALS AND METHODS

### 2.1 Description of the study area

This research was conducted in Harshin district, which is one of the eight districts in Faafen zone of Somali region (Figure 1). The district covers a total area of 5, 120 km^2^, and Harshin town (the district capital) is about 130 km southeast of the regional capital city, Jijiga. Meteorological reports indicate that the district has a moisture deficit due to limited amounts of rainfall (200–400 mm/year) and hot temperatures (mean average temperature 28°C). In the past, rainfall has been reliable in the district, but recently it is showing an erratic behavior with an increasing frequency of severe drought episodes (especially in 2015/2016) that have caused a lot of problems in the region. Annual evapotranspiration is also high in the 1,737–2,159 mm range (Berhanu, Melesse, & Seleshi, 2013). Thereby, according to the Köppen climate classification system, the district is categorized as hot semiarid climate. According to the latest census conducted in 2011, the population of Harshin district is about 92,901 people (90% rural and 10% urban) of which 51,096 (55%) are men and 41,805 (45%) are women. The inhabitants of the district belong entirely to the Somali ethnic group.

### 2.2 Background information: grazing pattern and practices

The predominant grazing pattern in the district is based on seasonal movement between wet and dry season pastures (transhumance). Traditionally, during wet seasons livestock are grazing near the herder's home area. However, in dry season (usually from July to October) cattle are moved to communal grazing areas and in the late dry season (October to late February) there is often mass mobility in search of better grass and water for animals.

An informal protection of communal grazing lands (area enclosure) in the area has been practiced since 2000. Before this period, the grazing areas within the districts were entirely communally owned. The new system of area enclosure for protecting grazing lands, however, is bringing a good result to stock grass for dry seasons. Thus, it is taken as a good adapting strategy (especially in drought years) and has been widely accepted by the community and is rapidly expanding in the district.

However, despite its acceptance and positive results, the regional government is opposing this activity and prohibiting pastoralist from doing so. The main reason is fears of shortage of communal grazing lands because of those enclosed grazing lands are apparently owned by the person who fenced it. Nevertheless, the pastoralists are still resistant and continue the practice and to avoid confrontation with government bodies they often use croplands as a buffer to enclose their grazing lands rather than the traditional wood and shrubs fences (Figure 2).

### 2.3 Types of data and methods of collection

Three types of data were used: field surveys, satellite imagery, and socioeconomic secondary data. Before the start of the rainy season, a preliminary field survey was conducted (May 15–20, 2018) to select appropriate study plots to measure above ground biomass (AGB) of grass. A total of 55 plots at two major sites were eventually selected and geo‐marked with GPS for further investigation, monitoring, and productivity measurement. To be considered for the study, a given plot needed to have a spatial coverage of at least 0.5 ha to conform to the pixel size of most of the freely available satellite images without being affected by reflection from nearby features or land cover. We used Garmin GPSMAP64 devices with a reported 3‐m accuracy to collect representative ground truth data for each land use and cover in the district. Thereby, a total of 170 Ground Control Points (GCP) were collected and used as a reference during image interpretation and classification accuracy assessment.

In addition to this, socioeconomic data related to human and livestock population were collected from Bureau of Finance and Economic Development of Jijiga and Atlas of the development profile of Harshin district. Informal interviews and discussion with key informants (e.g., local development agents and elders) were also carried out, particularly to obtain information about grazing patterns, area closures practices, and grass species names and their diversity. In addition, long‐term rainfall data obtained from Ethiopian metrological agency was analyzed in order to evaluate interannual rainfall variation of district.

### 2.4 Satellite image classification

Cloud‐free satellite images (acquired on May 11, 2018 by Sentinel‐2 sensors) were downloaded and processed to obtain the land use and cover map of the district. Sentinel‐2 data were acquired on 13 spectral bands in the visible and near‐infrared (VNIR) and shortwave infrared (SWIR) regions. Of the 13 spectral bands, four (B2, B3, B4, and B8) have a 10‐m resolution, and we used these bands for feature identification during classification. First, the downloaded images were geometrically corrected (UTM Zone 38N, datum Adindan, Spheroid‐Clark1888) to match the coordinate and projection system of GPS readings. Then, hybrid unsupervised and supervised classification was carried out. About 120 GPS points (20–30 samples for each class) were used for classification accuracy; thereby, our classification achieved overall accuracy of 92 percent. Finally, we clipped the areas covered by grass to estimate their productivity using average productivity value of measured AGB.

### 2.5 Selection of sampling sites and measurement of forage biomass production

Two sampling sites were selected in areas where exclosure (no human and animal intervention) is widely practiced. Animals were not allowed to enter to the site until we made biomass measurement, thanks to an agreement with the owners of the grazing land. Moreover, the two sites are ideally representative of the area productivity (low, medium, and high) and are considered to give average biomass productivity of the district. Site 1 represents high forage production area, whereas site 2 represents medium to low forage production area.

We measured AGB in 55 plots (13 high productive, 17 medium, and 25 low productive areas), which were previously selected in the two sites. The best time to measure was mid‐July (10–20) when the grass is naturally matured and has minimum moisture. We used a quadrant method (1 m^2^ area) to measure the biomass in the field. Quadrate was randomly thrown in sampling plot, and forage sample was cut at ground level with the help of a cutter. The fresh weight of forage sample was measured in the field with a scale. We then adopted a moisture correction factor value of 40%–50% (as suggested by Gay, Grisso, & Smith, 2009) to calculate the total dry matter (TDM) of the samples. The dry matter was converted into kilogram per hectare (kg/ha), and the proper use factor (PUF) was taken as 30% to calculate available forage (Guevara, Estevez, & Torres, 1996). Thereafter, dry matter (DM) biomass and livestock carrying capacity was determined following procedures described by De Leeuw and Tothill (1990).

### 2.6 Determine carrying capacity and stocking rate

Determining CC and stocking rates are crucial in rangeland management. The basic procedure for determining carrying capacity consists of calculating the total amount of forage at the end of the growing season, multiplying this by a correction factor (use factor) and then dividing by the average yearly feed requirements of a livestock unit (Hocking & Mattick, 1993).

Carrying or grazing capacity (CC) is described as the maximum number of animals that the rangeland can support without depleting the resources of the rangeland such as vegetation and soil (FAO, 1988). It can vary from year to year on the same area due to changes in forage production. However, all plants in the rangeland are not used by livestock, because some of them are not accessible to the animals, some are unpalatable and further losses occur due to animal trampling (Hocking & Mattick, 1993). Therefore, in order to come up with sustainable utilization of rangelands a proper use factor should be included, which varies according to different researchers and different situations from 30% in Southern Ethiopia (Cossins & Upton, 1987) to 45% in Tsavo, Eastern Kenya (Van Wijngaarden, 1985). Van Wijngaarden (1985) estimates that more than 55% of the grass covers should not be removed in one way or the other to keep the grasslands at healthy condition. So, to prevent grazing land from degradation and sustainable grassland use, at least 45% of the peak standing grass should be left at the beginning of the next rainy season. Other authors, Mugerwa (1992) and Kavana, Kizima, and Msanga (2005) used a proper use factor of 50% while Caltabiano (2006) proposed a factor of 30% for black spear grass and 20% for mulga pastures.

In this study, a use factor of 30%, as proposed by Cossins and Upton (1987) for Ethiopia, was adopted and used as consumable forage. We used the concept of tropical livestock unit (TLU) to calculate the CC of the range. The TLU is commonly taken to be an animal of 250 kg live weight, and daily feed intake per TLU was taken at 2.5% of the body weight (Sserunkuuma & Olso, 1998). Thus, TLU is used to bring all animal types under a common denominator and helps to compare feed needs for sheep, goats, calves, and other animals with those of dairy cows. Hence, we converted livestock of each kebele (lowest administration unit in Ethiopia) and the whole district into TLUs using FAO (1988) conversion factor.

Once the amount of forage yield and utilization rate was determined, the carrying capacity was calculated. The information can be used in two alternative ways: (a) to determine the number of heads a system can carry (TLU ha^−1^ year^−1^) or (b) to determine how many areas a specific herd can graze in the system (ha TLU^−1^ year^−1^). Thereby, we used the following equation to determine the carrying capacity of grazing lands in Harshin district (FAO, 1988):

(1) $$\begin{array}{c} \text{CC}\left(\text{kg}\;\text{of}\;\text{live}\;\text{weight}\;{\text{ha}}^{-1}\;{\text{year}}^{-1}\right)\\ =\text{potential}\;\text{yield}\;\text{of}\;\text{TDM}*\text{usable}\;\text{rate}\;\left(30\%\right)*100/2.5*365 \end{array}$$

Thus, the result can be explained both as TLU ha^−1^ year^−1^ and ha TLU^−1^ year^−1^ depending on the interest of the interpreter.

On the other hand, stocking rate is the number of animals on a grazing land for a specified time period and is usually expressed in TLU ha^−1^ year^−1^. Similar to CC calculation, we applied 30% consumable rate on the potential yield and calculated the stocking rate using the following formula:

(2) $$\text{Stocking}\;\text{rate}\;\text{for}\;\text{the}\;\text{year}\;\left(\text{TLU}\;{\text{ha}}^{-1}\;{\text{year}}^{-1}\right)=\text{TLU}/\text{total}\;\text{grazing}\;\text{area}$$

### 2.7 Determination of equilibrium and nonequilibrium rangeland systems

In equilibrium environments where rainfall is relatively stable, grazing dynamic is dependent on the balance between carrying capacity and stocking rates; therefore, it is appropriate to calculate carrying capacities and use them to define sustainable livestock populations (Okayasu, Nakamura, & Takeuchi, 2008). In contrast, nonequilibrium environments are primarily characterized by fluctuations in parameters such as rainfall and the resulting fluctuations in plant biomass (primary production) and the corresponding carrying capacity (Vetter, 2005). Therefore, recognizing the difference between equilibrium and nonequilibrium environments is important mainly because of the most appropriate management strategies are considerably different (Scoones, 1999). Interventions based on the equilibrium systems focus on reducing stocking rates and increasing steadiness, while the nonequilibrium paradigm encourages opportunistic stocking strategies and promote mobility (vetter, 2005). Therefore, in order to determine the rangeland system (equilibrium or nonequilibrium) of Harshin district, we evaluated long‐term interannual rainfall fluctuations and calculated coefficient of variation in the district.

### 2.8 Data analysis

The rangeland productivity and livestock data were analyzed by means of Microsoft Excel program to generate descriptive statistics. Besides, we used ERDAS2010 and Arc GIS10 software to analyze remote sensing data and prepare land use and cover map.

## 3 RESULTS AND DISCUSSIONS

### 3.1 Current land use/cover types of the district: operational use and definitions

**Figure 3:**
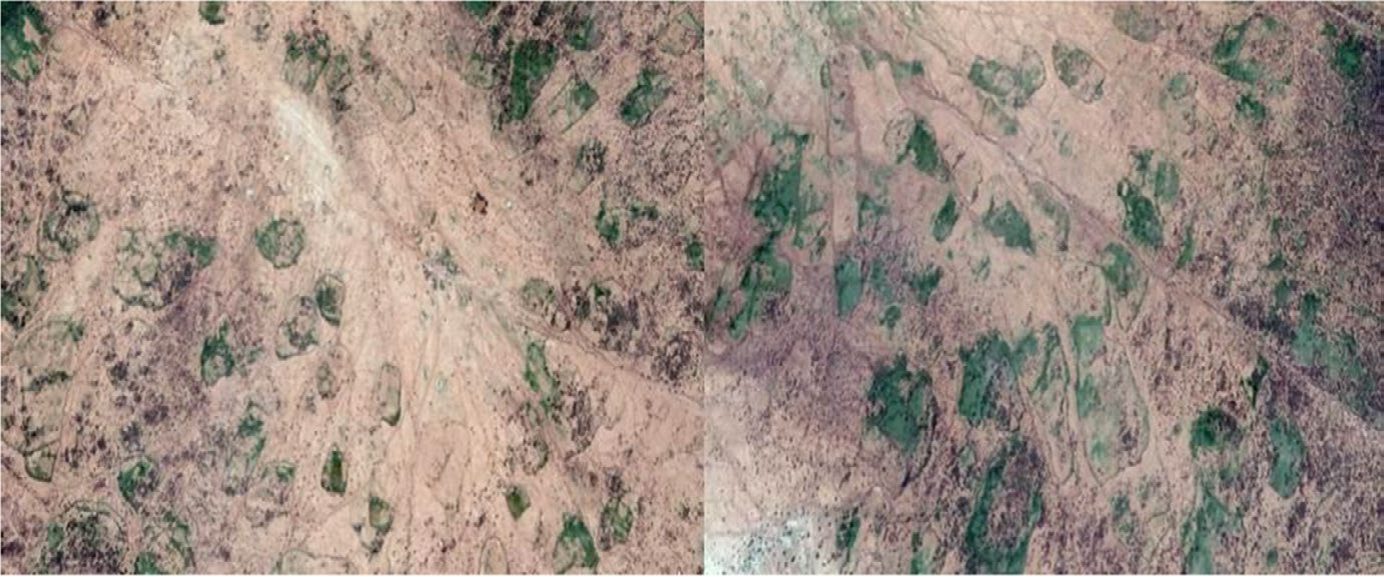
Land use and cover map of Harshin as derived from Sentinel 2 (June 2018)

The dominant LULC that we identified in the study area, include woodland (35.5%), shrubs (28.3%); grassland (10.6%), and bareland (25.5%) (Figure 3 and Table 1). Other land uses such as settlement and farmlands are not included due to their smaller proportions.

**Table 1:** Land use and cover types of Harshin district (2018) with their operational definitions and area coverage

Land cover types	Area coverage		Operational Land use	Brief description of each land cover
	Hectares	%		
Grassland	51,015.8	10.6	Grazing to animals (shoats and cattle)	All areas covered mainly by different grass species and small sized an herbaceous plant which is used as a natural pasture. More often tree, shrub, and bush are very scattered
Shrubland	136,438.7	28.3	Browsing to mainly goats and dromedaries	Land covered with sparse woody acacia plants mixed with shrubs, bushes, and grasses
Woodland	170,910.7	35.5	Browsing to animals (mainly dromedaries); source of fuel (charcoal and fire wood)	Land with woody species cover >50% (height ranges 5–20 m) and mostly dominated by acacia
Bareland	122,902.3	25.5	Barren land with no economic value	Areas mainly with no vegetation cover and to some extent very scattered Acacia tree or nonvegetated areas, or areas with very little vegetation cover and bedrock exposed to surface
Total	481,267.5	100.0		

The region has browse‐rich shrubland (thorny bush) that is edible to dromedary and goats, as well as massive grasslands plains for sheep and cattle grazing. Some areas are rich in trees that produce gums and resins. The description and operational use of each land cover is presented in Table 1.

### 3.2 Livestock population of the district

Pastoralism is the dominant form of survival in the district providing livelihood to over 70% of the population. Pastoralists rear all types of livestock such as dromedary, cattle, sheep, and goats in the district. Agropastoralists are very few. They grow crops (sorghum and maize) in parallel with animal rearing.

The total livestock population of the district is about 1,174,459 of which 573,515 (48.82%) are sheep, 399,815 (34.03%) are goats, 134,090 (11.41%) are dromedaries, 48,645 (4.14%) are cattle, and 17,343 (1.48%) are donkeys (BOFED, 2012). The remaining 1,051 are mules, horses, and poultry. In proportion, sheep and goats are considered to be the first‐rate livestock species followed by dromedary and cattle (Table 2).

**Table 2:** Livetock types and population at different kebeles of Harshi district

No	Kebele	Cattle	Goats	Sheeps	Donkeys	Dromedaries	Poultry	Mules	Horses
1	Harshin	1,602	11,413	5,964	292	1,986	89	0	0
2	K/Ramaale	4,812	27,021	41,185	966	4,619	0	0	0
3	Madaweyn	1,614	15,305	15,250	925	10,530	40	0	0
4	D/Weyne	12,311	20,384	31,172	2,137	11,363	0	0	0
5	Lafa Islamod	8,824	17,365	19,357	1,285	10,299	451	4	32
6	Farah Liban	5,703	16,129	86,177	1,602	7,781	0	0	5
7	Darbiga	5,257	42,951	61,131	1,063	10,810	324	23	24
8	Bali'ase	855	29,384	34,940	756	4,976	0	0	0
9	Afufle	0	38,348	32,824	1,069	11,250	0	0	0
10	Lankerta	745	29,079	34,557	740	4,776	56	0	0
12	Aran'are	4,600	25,000	34,720	2,030	10,650	0	0	3
14	Bale'gubadle	636	10,472	14,831	269	740	0	0	0
16	K/Hashim	1,671	16,796	69,858	2,109	24,893	0	0	0
17	A/Ahmed	15	100,168	91,549	2,100	19,417	0	0	0
	Total livestock	48,645	399,815	573,515	17,343	134,090	960	27	64
	TLU factor	0.7	0.1	0.1	0.5	1	0.01	0.7	0.8
	Total TLU	34,051.5	39,981.5	57,351.5	8,671.5	134,090	9.6	18.9	51.2

### 3.3 Productivity of rangelands

**Figure 4:**
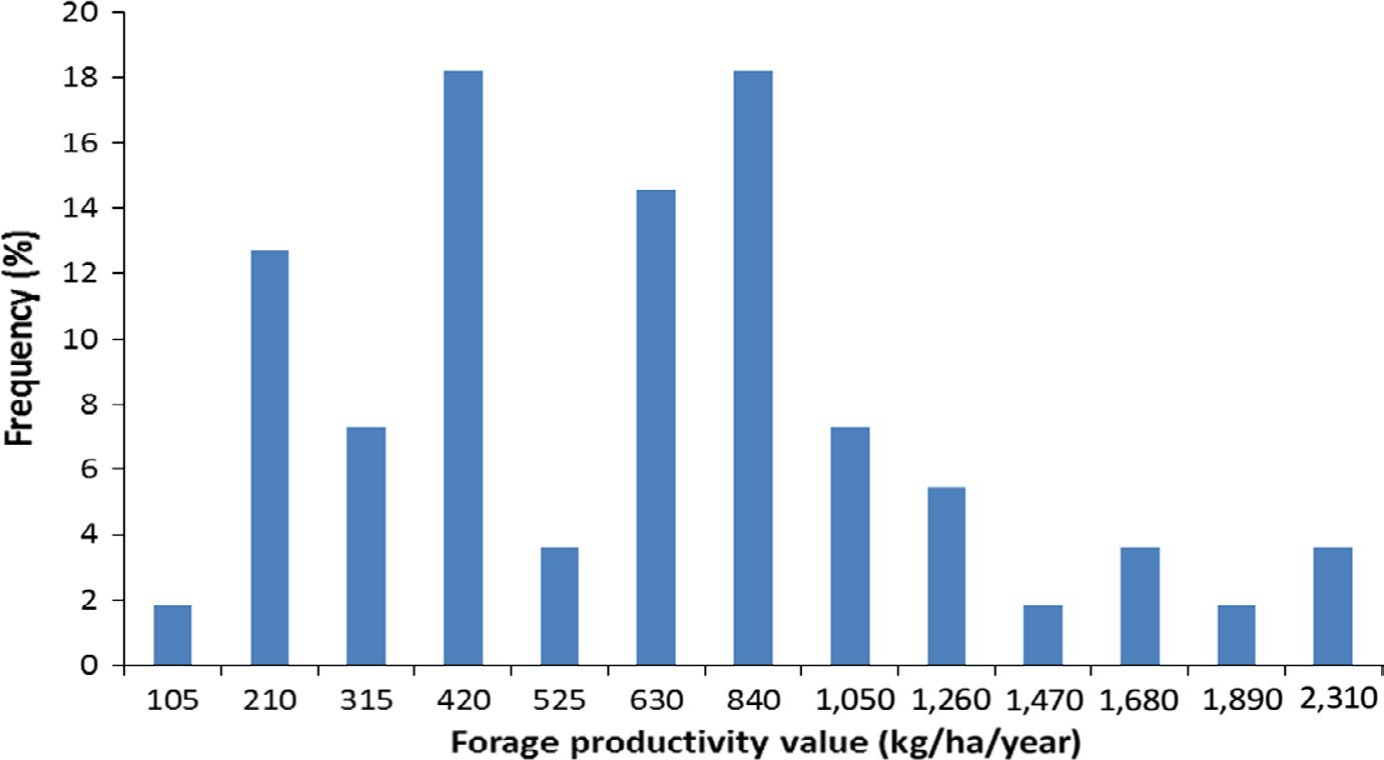
Histogram of measured forage biomass data

We measured above ground biomass (AGB) of grass‐forage at 55 different plots and summarized the result in Table 3. The result shows that the minimum and maximum forage produced were 105 and 2,310 kg/ha, respectively (Figure 4), whereas the average productivity of the district is 742.6 kg/ha. Then, we used this average productivity value from the plots (742.6 kg/ha) and grazing areas (derived from satellite) to estimate the total dry matter (TDM) of the district.

**Table 3:** Species diversity, productivity and carrying capacity of rangelands in Harshin district, Somali region, Ethiopia (July 10–20, 2018)

Plot No.		Level of productivity	GPS coordinates		Height and weight of dry matter					Carrying capacity (CC)		Dominant grass species	
			X‐reading	Y‐reading	Grass height (m)	Mean DM (g/m^2^)	DM (kg ha^−1^ year^−1^)		DM (ton ha^−1^ year^−1^)	TLU ha^−1^ year^−1^	ha TLU^−1^ year^−1^	Local name	Scientific name
1		High	378,660	981,063	0.6	126	1,260		1.26	0.6	1.8	Baldhoole	*P. maximum*
2		High	378,741	981,111	1.0	168	1,680		1.68	0.7	1.4	Baldhoole	
3		High	378,878	981,127	1.0	231	2,310		2.31	1.0	1.0	Baldhoole	
4		High	378,924	981,132	0.9	231	2,310		2.31	1.0	1.0	Dhikil	*H. contortus*
5		Medium	378,983	981,164	0.5	84	840		0.84	0.4	2.7	Xarfo	*C. virgata*
6		High	379,025	981,167	1.0	126	1,260		1.26	0.6	1.8	Baldhoole	*P. maximum*
7		Medium	379,061	981,145	0.5	63	630		0.63	0.3	3.6	Xarfo	*C. virgata*
8		High	379,093	981,339	0.9	189	1,890		1.89	0.8	1.2	Dhikil	*H. contortus*
9		High	379,087	981,384	1.0	168	1,680		1.68	0.7	1.4	Dhikil	
10		High	357,652	963,458	0.9	126	1,260		1.26	0.6	1.8	Dareemo	*C. aucheri*
11		Medium	357,761	963,448	0.7	84	840		0.84	0.4	2.7	Xarfo	*C. virgata*
12		Medium	357,804	963,437	0.8	84	840		0.84	0.4	2.7	Xarfo	
13		High	357,840	963,468	0.8	147	1,470		1.47	0.6	1.6	Dareemo	*C. aucheri*
14		Medium	357,905	963,489	0.7	84	840		0.84	0.4	2.7	Dareemo	
15		Medium	356,990	964,139	0.8	84	840		0.84	0.4	2.7	Dareemo	
*Continue*…													
43	Low		357,652	963,680	0.5	31.5	315		0.315	0.1	7.2	Dareemo	*C. aucheri*
44	Medium		357,615	963,714	0.5	63		630	0.63	0.3	3.6	Ciirdhuuq	*C. ciliaris*
45	Low		357,582	963,697	0.4	21		210	0.21	0.1	10.9	Ciirdhuuq	
46	Low		357,559	963,736	0.5	42		420	0.42	0.2	5.4	Ciirdhuuq	
47	Low		357,530	963,712	0.4	42		420	0.42	0.2	5.4	Ciirdhuuq	
48	Low		357,388	963,768	0.4	31.5		315	0.315	0.1	7.2	Ciirdhuuq	
49	Low		357,345	963,795	0.5	31.5		315	0.315	0.1	7.2	Dareemo	*C. aucheri*
50	Low		357,314	963,822	0.5	21		210	0.21	0.1	10.9	dareemo	
51	Low		357,203	963,854	0.6	52.5		525	0.525	0.2	4.3	Dareemo	
52	Low		357,141	963,916	0.5	42		420	0.42	0.2	5.4	Xarfo	*C. virgata*
53	Medium		357,060	963,936	0.5	84		840	0.84	0.4	2.7	Baldhoole	*P. maxmum*
54	Low		357,018	963,946	0.4	21		210	0.21	0.1	10.9	xarfo	*C. virgata*
55	Low		356,945	964,008	0.6	52.5		525	0.525	0.2	4.3	Ciirdhuuq	*C. ciliaris*
Average value					0.6	74.3		742.6	0.7	0.3	4.9		
Min. value					0.4	10.5		105.0	0.1	0.0	1.0		
Max.value					1.0	231.0		2,310	2.3	1.0	21.7		

The biomass measurements from the plots were categorized into three levels of productivity: high (>1,000 kg/ha); medium (500–1,000 kg/ha); and low (<500 kg/ha). Accordingly, 24%, 32%, and 43% of the plots were representative of high, medium, and low productivity areas, respectively. There is not much variation in slope (0%–5%) and elevation (1,271–1,341 m.a.sl) among the plots where the biomass was measured. The height of grasses ranges between 0.4 and 1 m and shows marked variation among the different species (Table 3).

It should be noticed that forage biomass of an area is highly dependent on annual rainfall amount. Therefore, the productivity measured and reported here should be interpreted with caution; it is expected to be valid in an average annual rainfall amount of around 300 mm (which is the case of this year—2018—in the area). If the annual rainfall amount has variation compared to 2018, the productivity that we reported in Table 3 will be subjected to some variation (increase or decrease). Fortunately, the rainfall amount of 2018 (280 mm) is close to the average annual rainfall of the area, so the result can be taken as average productivity of the district. The annual rainfall amount of the area ranges between 200 and 400 mm (except drought years which happen at least once in a decade), and the mean annual average is 300 mm. In line with this, Tucker, Praet, Boerwinkle, and Gaston (1983) reported that during the favorable years of 1980–1982 (about 350 mm), average TDM was 1.0–1.3 t/ha and CC was calculated at about 5 ha/TLU (20 TLU/km^2^); however, in 1983, when rainfall was very low (about 100 mm), TDM was below 0.2 t/ha and caused a serious shortage of feed and a high rate of livestock mortality. In another study (conducted at Mongolia), Bat‐Oyun, Shinoda, Cheng, and Purevdorj (2016) reported that better TDM was obtained in wet conditions: 2008 (139.6 mm; 147.5 ± 26.5 g/m^2^); 2004 (137.6 mm; 93.0 ± 11.9 g/m^2^); 2005 (69.8 mm; 87.1 ± 9.0 g/m^2^); and 2006 (72 mm; 44.9 ± 6.2 g/m^2^). This indicates that biomass is highly reliant on annual rainfall amount of the area and hence our estimation for Harshin is not a fixed value rather might be changed depending on the rainfall amount of the year.

When our finding at Harshin district (forage biomass productivity) is compared with similar research of other Africa countries, it is within the range of Rains and Kassam (1979) reports (1.7–0.5 t DM/ha) who estimated TDM values for West Africa at 400 mm/year. However, it is lower than van Wijngaarden (1985) estimation for Eastern Kenya at 400 mm/year (2.3 t DM/ha) and Dye and Spear (1982) estimation for Zimbabwe at 400 mm (1.7 t DM/ha). This implies that the district is found at medium level of forage production. Nevertheless, with continuation of area closure management system (that creates perennial grass cover) the TDM is expected to rise. Because, the main impact of area closure management system on forage production comes at the later age than its beginning. Furthermore, the observed rainfall amount in the district is lower (280 mm) than the other African countries (400 mm) that are compared; consequently, it has contribution for the biomass of the district to be relatively lower than most of the other countries.

### 3.4 Species diversity in the rangelands

**Figure 5:**
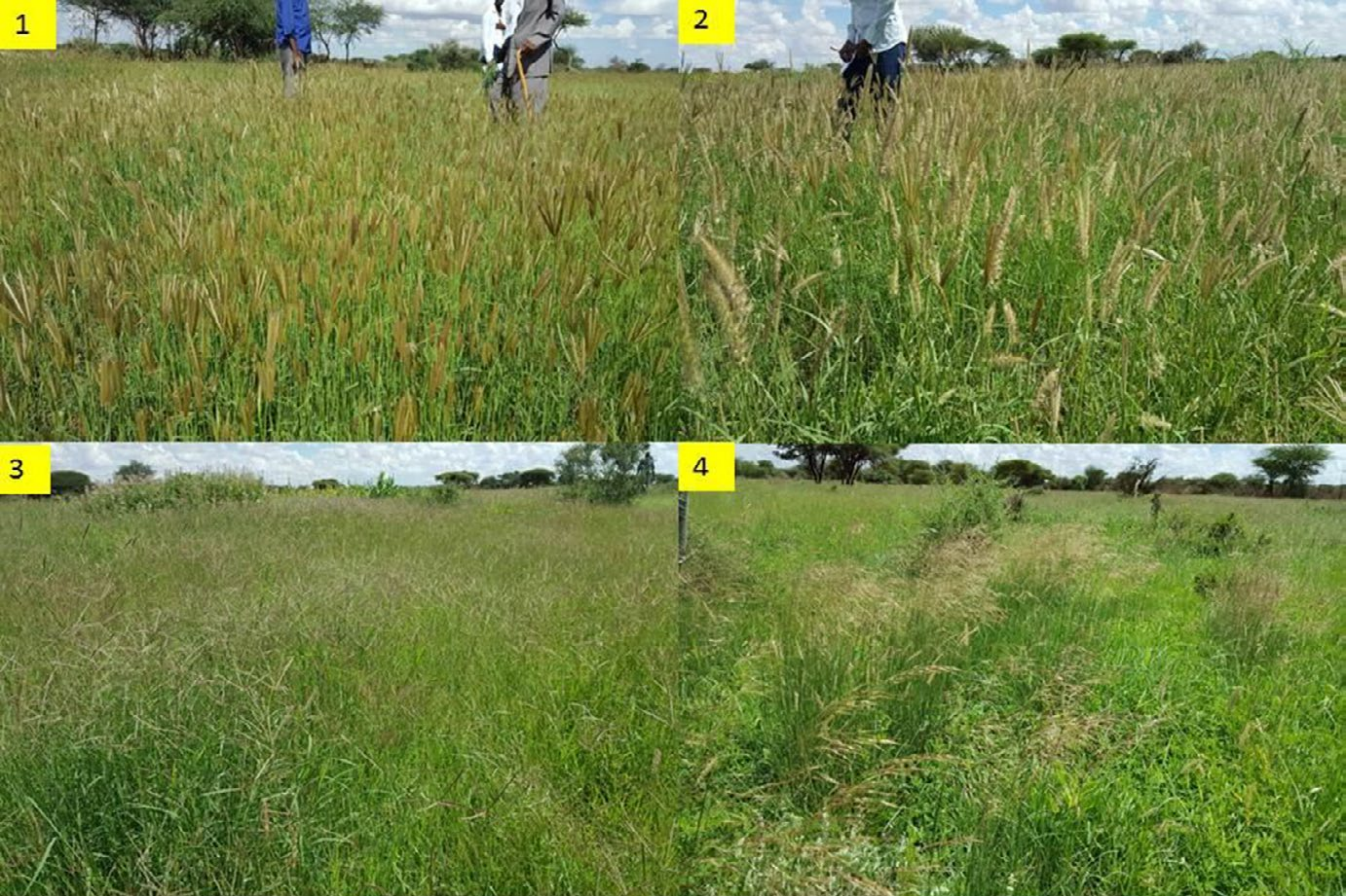
The dominant grass species at grazing lands of the areas: (1) Baldhoole (*Panicum maximum*); (2) Ciirdhuuq (*Cenchrus ciliaris*); (3) Xarfo (*Chloris virgate)*; (4) Dareemo (*C. aucheri*)

There are different grass species in the rangeland but the most dominant ones that we widely observed and were identified by the pastoralists include dareemo, baldhoole, dhikil, xarfo, and ciirdhuuq (Figure 5 and Table 3). They are also known by their scientific names as *Chrysopogon aucheri*,* Panicum maximum*,* Heteropogon contortus*,* Chloris virgata*, and* Cenchrus ciliaris*, respectively.

The different species have different range of grass height and weight. Thus, in average grass height and weight *H. contortus* and *P. maximum* rank equally (first) followed by *C. virgata*, *C. aucheri*, and *C. ciliaris* (Table 3). However, according to our interview with farmers, in terms of animals preference (grass palatability), and impact to their weight (meat) and milk production *P. maximum* ranks first followed by *C. aucheri*, *Cenchrus ciliaris*, and *C. virgata*. Hence, our surveys and discussion with pastoralists reveals that they would like to have more *C. aucheri* grass in their grazing lands rather than other species for simple reason of maximizing their meat and milk production.

### 3.5 Carrying capacity of rangelands

In study site, plot‐based CC values range between 0 and 1 TLU ha^−1^ year^−1^ (1–21.7 ha TLU^−1^ year^−1^) with an average value of 0.3 TLU ha^−1^ year^−1^ (4.9 ha TLU^−1^ year^−1^) (Table 3 above). Our result compares reasonably well with 5.5 ha/TLU (0.2 TLU/ha) for East Africa given by Pratt and Gwynne (1977) for the same annual rainfall. However, it is relatively lower than Byenkya (2004) and Mugerwa (1992) reports of the same region (2.27 and 1.63 ha/TLU, respectively) and Hocking and Mattick (1993) results for wooded grasslands of Tanzania (2.5–3.5 ha/TLU). The comparison with other similar studies indicates that the rangeland in Harshin is in relatively fair to poor condition. However, the district has potential for browsing of shrubs and trees that can supplement grazing but that were not included in the study. The CC value reported here might be a conservative and underestimate the actual CC potential of the district especially because dromedaries and goats equally browse and graze.

### 3.6 Stocking rates and sustainability of grazing lands in the district

The sustainability of the grazing land in the district was evaluated through comparing the CC with its existing stocking rate and observing if they were balanced or not. The actual stocking rate of the district was estimated taking into account the total TLU and available animal forage in the district. Thus, the existing stocking rate has become 5.4 TLU ha^−1^ year^−1^ (0.18 ha TLU^−1^ year^−1^), which is much higher than its carrying capacity (0.3 TLU/ha or 4.9 ha/TLU). In line with this, Pratt and Gwynne (1977) suggested 4‐hectare lands as optimal area for supporting one TLU annually in the East African semiarid rangelands.

**Figure 6:**
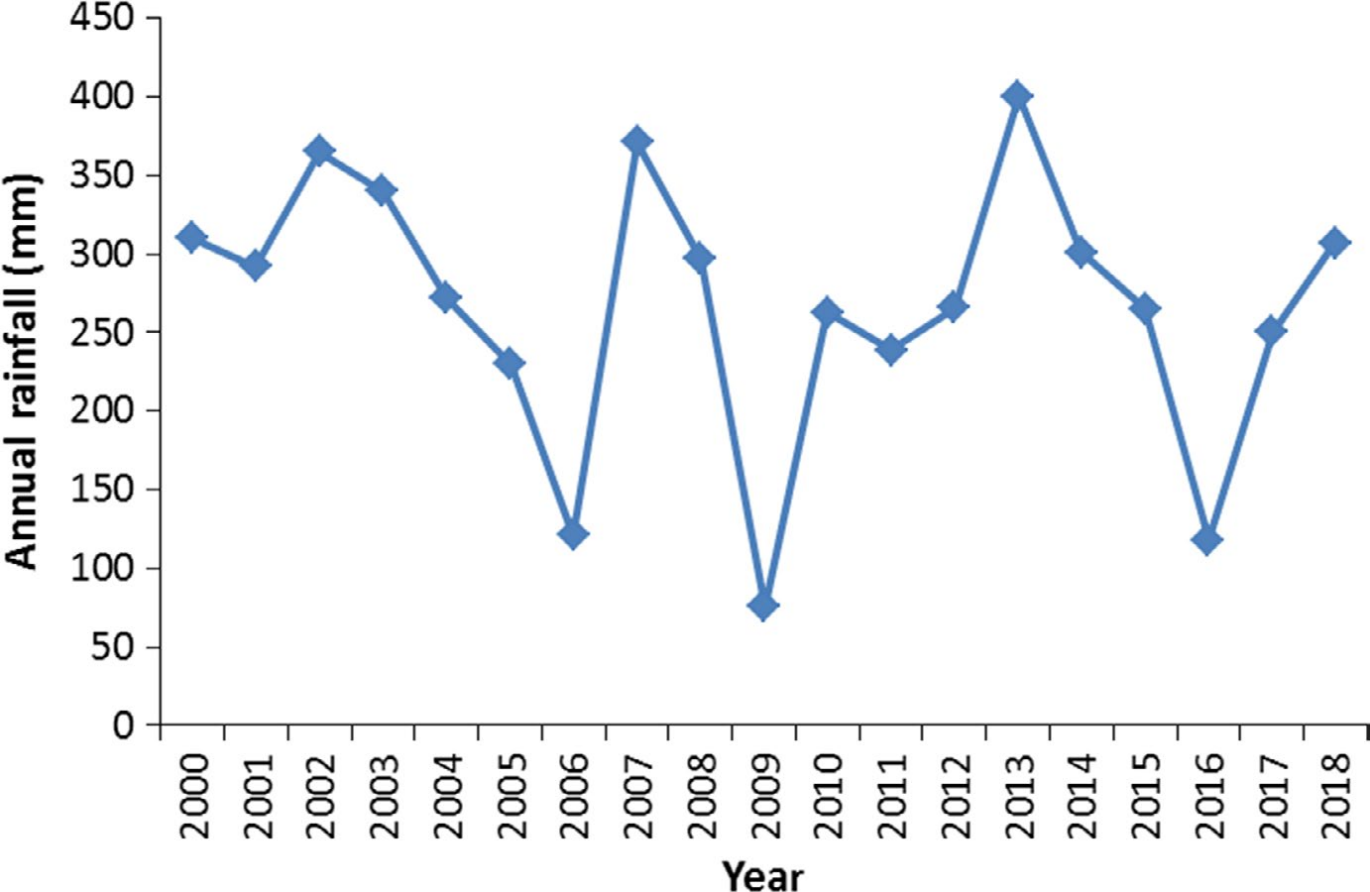
Interannual rainfall variability of Harshin District

The meteorological data of the last 18 years (2000–2018) indicate no significant variation (except the drought years) among interannual rainfall (Figure 6). The mean annual rainfall and the calculated CV (excluding the extreme drought years) has become 298 mm and 16.5%, respectively. Thus, when the drought years are excluded the rangeland becomes near‐equilibrium system because the nonequilibrium dynamics predominate in areas where rainfall CV exceeds 33% (Ellis, Coughenour, & Swift, 1993; Vetter, 2005). Rangeland management in an equilibrium system (such as Harshin district) is based on Clementsian succession theory (Clements, 1916) and is implemented by controlling livestock numbers below the level that would cause degradation of the ecosystem (i.e., the carrying capacity), while maximizing the productivity of the livestock (Caughley, 1979). On the other hand, Engler et al. (2018) and Vetter (2005) reported that management of African rangelands has generally followed the equilibrium model and the assumption that high number of livestock can lead to overgrazing. Thereby, government interventions include destocking schemes, conversion of communal areas into individually tenures, and settling of nomadic pastoralists into group farmers (resettlements) (Archer, Hoffman, & Danckwerts, 1989; Ellis & Swift, 1988; Rohde, Hoffman, & Cousins, 1999).

In Harshin district, for the past decades, occurrence of drought remains to be the major disaster causing huge damages to the populations and economy of the district. According to Ethiopian Metrological Agency (EMA) and local sources, the area has experienced 15 major droughts during the period between 1960 and 2017. The droughts that occurred in 1973–1974, 1984, 1991, 2006/2007, 2009/2010, and 2016/2017 were most intense and widespread. Therefore, the recommended management to Harshin district should be integration of equilibrium and nonequilibrium system. Thereby, during the drought years the strategic management should be opportunistic stocking and mobility, and if the drought is worse the government should support them to destock and restock animals in the available marketing system. Because drought risks are minimized not by maintaining conservative stocking rates, but rather by allowing livestock numbers to increase in wet years. While, in normal rainfall years the livestock numbers (stocking rate) should not exceed the calculated carrying capacity (number here) in order to sustainably use the ecosystem. In agreement with this, recent studies recommended that most arid and semiarid rangeland systems encompass elements of both equilibrium and nonequilibrium at different scales, and that management needs to take into account temporal variability and spatial heterogeneity (Briske, 2017; Mihertu, Xinwen, & Yong‐dong, 2018; Vetter, 2005).

### 3.7 Conclusions and recommendations

We used field surveys, satellite imagery, and secondary data to estimate forage biomass productivity, carrying capacity, and stocking rate of Harshin district.

We conducted biomass measurement on 55 plots having different level of productivity. The result shows that the minimum and maximum forage produced were 105 and 2,310 kg/ha respectively; whereas, average productivity of the district is 742.6 kg/ha. The calculated carrying capacity (CC) value for the district ranges between 0 and 1 TLU ha^−1^ year^−1^ (1–21.7 ha TLU^−1^ year^−1^) with average value of 0.3 TLU ha^−1^ year^−1^ (4.9 ha TLU^−1^ year^−1^); while, the existing stocking rate has become 5.4 TLU ha^−1^ year^−1^ (0.18 ha TLU^−1^ year^−1^). It is apparent that there is a great disparity between the observed stocking rate and the carrying capacity of the production system because the existing stocking rate has grazing pressure in excess of 5.1 TLU/ha (~7.2 cattle or 51 sheep/ha). If this trend continues, there will be overgrazing pressure and expansion of land degradation, which will have immense consequence on the sustainable use of grazing lands in the future.

Therefore, urgent reaction from the government, policy, and decision maker is required. They are supposed to ease the existing grazing pressure and look for new grazing sites taking into account human and animal population growth in the future. The new trend of protecting grazing lands (area closure) is bringing a good result to secure grass to animals. Hence, we strongly suggest regional and federal government to support the practice in the district and expand it to other zones as a coping mechanism of drought and grazing land management.

Calculated interannual rainfall variation is 16.5% which implies that rangeland is a subsistence equilibrium system, and degradation in the area is caused by overstocking. On the other hand, occurrence of drought remains to be the major disaster causing huge damages to the populations and the economy of Harshin district. Therefore, the recommended management to Harshin district should be integration of equilibrium and nonequilibrium system. During the drought years the strategic management should be opportunistic stocking and mobility, and if the drought is worse the government should support them to destock and restock animals in the available marketing system. While, in normal rainfall years the livestock numbers (stocking rate) should not exceed the calculated carrying capacity (0.3 TLU ha^−1^ year^−1^) in order to sustainably use the ecosystem.

## CONFLICT OF INTEREST

None declared.

## AUTHOR CONTRIBUTIONS

All authors made contribution at different stages of this research. DTM was a leader of the research and hence designed, collected and analyzed field data, and interpreted the result. He also wrote the draft manuscript. MM and DY made significant contribution in the design of the study, field data collection and provide comments on the draft manuscript. All authors read and approved the final manuscript.

## Data Availability

The data that support the findings of this study are available from the corresponding author (Derege T. Meshesha) and can be accessible through the following link: https://doi.org/10.5061/dryad.c2fqz6144
